# China's lunar and deep space exploration: touching the moon and exploring the universe

**DOI:** 10.1093/nsr/nwz120

**Published:** 2019-08-16

**Authors:** Weijie Zhao, Chi Wang

**Affiliations:** 1 NSR news editor based, Beijing; 2 National Space Science Center, Chinese Academy of Sciences

## Abstract

The Chinese lunar probe Chang'e-4 (CE-4) landed in the Von Kármán crater within the South Pole–Aitken (SPA) basin on the far-side of the Moon on 3 January 2019. Following this, the moon rover Yutu-2 separated from the CE-4 lander and started its travels and exploration on the far-side of the Moon. Before this landing, humans had remotely observed the far-side of the Moon with lunar satellites. However, it was the first time that a man-made spacecraft had landed there and actually left behind wheel prints belonging to humanity.

Since China's Lunar Exploration Project (CLEP), or Chang'e Project, started in 2004, China has accomplished the first two steps of its three-step plan of ‘Orbiting, Landing and Returning’. CE-3 and CE-4 landed successfully on the near-side and far-side of the Moon, respectively. In the near future, CE-5 will land again on the near-side of the Moon and take lunar rock and soil samples back to Earth, thus completing the three-step plan of CLEP. In April 2019, *National Science Review* (NSR) interviewed three key figures of CLEP: CLEP Chief Engineer Weiren Wu (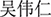), the first CLEP Chief Scientist and CLEP senior consultant Ziyuan Ouyang (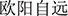), and CLEP third phase Vice-Chief Engineer, CE-4 Ground Research and Application System Director Chunlai Li (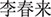). They talked about the scientific expectations and future plans of China's lunar and deep space exploration.

## THE ONGOING CE-4 MISSION

**NSR:** How is the current condition of the CE-4 lander and Yutu-2?

**Wu:** Their condition is rather good. The design lifetime of the CE-4 lander and Yutu-2 (the rover) is six and three lunar days, respectively. (A lunar day lasts for about 28 Earth days, including a lunar night of about 14 Earth days.) Now they are beginning their fifth lunar day of work, which means that Yutu-2 has already exceeded its design life and the lander is approaching its design life. Yutu-2 has travelled on the moon for about 180 m. The instruments on the lander and Yutu-2 are operating well and collecting data about the topography, geological structure, mineral composition and space environment of the far-side of the Moon.

**NSR:** What are the major technological advances for landing on the far-side of the Moon?

**Wu:** There are two major changes compared with CE-3. Firstly, since the direct communication between the Earth and the far-side of the Moon is blocked by the Moon body, we need to establish a communication path through a relay satellite. The relay satellite Queqiao was launched in May of 2018. It reached and stabilized around the Earth–Moon Lagrangian point 2 and is able to transmit signals between the Earth and the far-side of the Moon.

**Figure 1:**
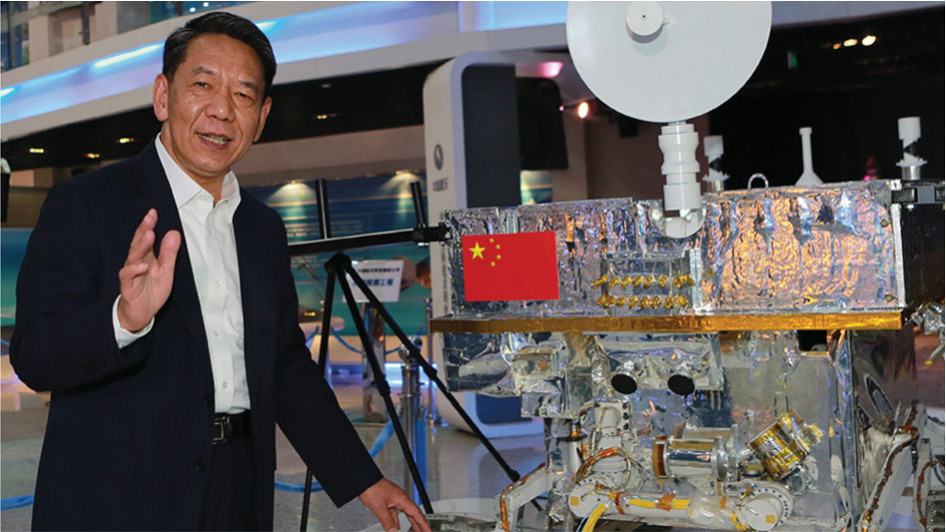
CLEP Chief Engineer Weiren Wu with a model of Yutu, the rover of CE-3 *(Courtesy of Weiren Wu)*.

Secondly, CE-4 adopted a new landing procedure. The near-side of the Moon has large plains and is relatively easy to land on so that CE-3 can slide down at a certain angle. However, the topography of the far-side of the Moon is more complex with many lunar mountains and lunar craters. CE-4 took a near-vertical landing routine in order to avoid hitting the mountains. Actually, we spent a lot of time determining the landing point of CE-4. Its pre-selected landing area is very small—as small as one-eighth of that of CE-3.

**NSR:** Why are we interested in the far-side of the Moon? What are the major scientific missions of CE-4?

**Ouyang:** CE-4 has three major scientific tasks, all of which cannot be achieved without landing on the far-side of the Moon. The first one is to collect long-wavelength signals from the Sun, the solar system, the Milky Way Galaxy and other parts of the universe. Due to interference from the noise generated by the Earth's ionosphere, we cannot obtain signals with wavelengths longer than 1 m on the Earth or on the near-side of the Moon. With the Moon body blocking the interference of the Earth's ionosphere, it is possible to collect signals with wavelengths as long as 10 m, 100 m, or 1 km on the far-side of the Moon, allowing us to learn about the Sun and the universe using information in this new wavelength band. CE-4 carried a low frequency spectrometer to collect these signals.

**Figure 2:**
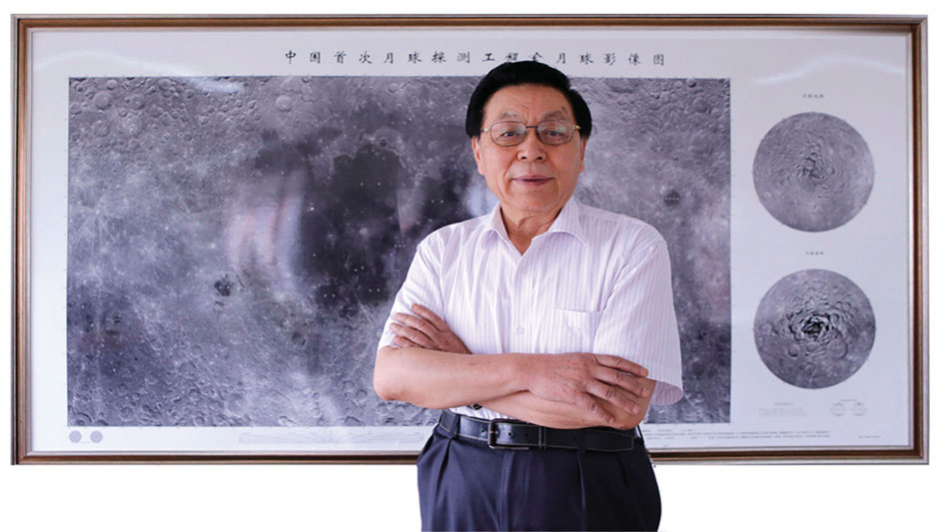
The first CLEP Chief Scientist and CLEP senior consultant Ziyuan Ouyang with a lunar map captured by CE-2 *(Courtesy of Ziyuan Ouyang)*.

The second task is to explore the early geological history of the Moon. The Moon is now a ‘dead celestial body’ with no magma activity. However, it was active between 3 and 4.5 billion years ago, when there were abundant magmatic eruptions, volcanic activities and tectonic movements with new rocks formed. On the near-side of the Moon, we have learnt much about the Moon's history between 3 and 4 billion years ago, but not beyond 4 billion years because the rocks formed at that time were buried deep under the lunar surface or covered by later volcanic lava flows. However, about 4 billion years ago it is believed that a celestial body hit the far-side of the Moon. Its shock wave created a huge basin of 2480 km in diameter—the SPA basin in which CE-4 landed. This event peeled off the surface rocks and exposed many rocks formed before 4 billion years ago. By landing in the SPA basin, we should be able to study older lunar rocks and learn about the earlier lunar history. We installed lunar penetrating radar as well as a visible and near-infrared imaging spectrometer on Yutu-2 to explore the shallow underground structures and the mineral composition of lunar rocks in the SPA basin.

The third scientific task of CE-4 is to detect the space environment on the far-side of the Moon. We suppose that the space environment on the far-side would be similar to that of the near-side. But we need the actual data to confirm this. Two of our international payloads are targeting this task, including the Lunar Lander Neutrons and Dosimetry provided by German scientists on the CE-4 lander, and the Advanced Small Analyzer for Neutrals developed by Swedish scientists on Yutu-2.

**Li:** The first batch of data from CE-4 has returned. We have begun data analysis and are preparing to publish the results.

**NSR:** Will the CE-4 data be open to the international community?

**Li:** The data will be open to Chinese scientists six months after we obtain the data, and will be open to the international community another six months later. This is the common practice throughout the world. And for the international payloads, we will immediately share the data with our partners.

## FUTURE PLANS

**NSR:** Will China continue with the exploration of the far-side of the Moon?

**Wu:** We do not have further plans in the short term. So CE-4 and its data are very valuable. It is the first time and will be the only time in the short term for the far-side landing on the Moon, although this might be done again in the distant future.

**NSR:** What is the future plan of China's lunar and deep space exploration?

**Wu:** Considering costs and risks, the primary direction of our space exploration is robotic exploration. We will launch CE-5 in the near future. It will land on the near-side of the Moon and take back lunar samples. After that, we plan to build a lunar research station at the south pole of the Moon. There are two reasons for choosing the south pole. Firstly, there can be half-year's continuous solar illumination on the south pole, similar to the Earth's poles, so that the scientific instruments will be able to work continuously for a long period. Secondly, it has been proposed that in the deep moon pits at the lunar poles, where no sunshine can reach, there may be water ice remaining. We want to further detect and confirm this possibility.

We also have plans for deep space exploration. China's first Mars probe will be launched in the not too distant future. Plans for Jupiter and asteroid exploration are also under study. We also want to explore the boundary of the heliosphere where the solar wind ends. This boundary is about 100 astronomical units (AU; 1 AU = 149 597 870 700 m ≈ the distance from the Earth to the Sun) away from the center of the solar system. It will take a man-made probe 20 to 30 years to reach there. We hope to be able to do that.

**NSR:** What is the cost of CLEP? Will the government continue to support the space exploration?

**Wu:** Lunar and deep space exploration is the will of the country, and is the only way for mankind to find extraterrestrial habitable planets in the future, so the government will definitely support it. Within the past 15 years since 2004, the annual average investment in CLEP accounts for only 0.003% of China's current annual GDP. It is not expensive compared with projects such as the Apollo program of the United States.

We plan to build a lunar research station at the south pole of the Moon.

—Weiren Wu

## ORGANIZATION AND COOPERATION IN THE BIG PROJECT

**NSR:** How many people are involved in CLEP? How is the project organized?

**Figure 3:**
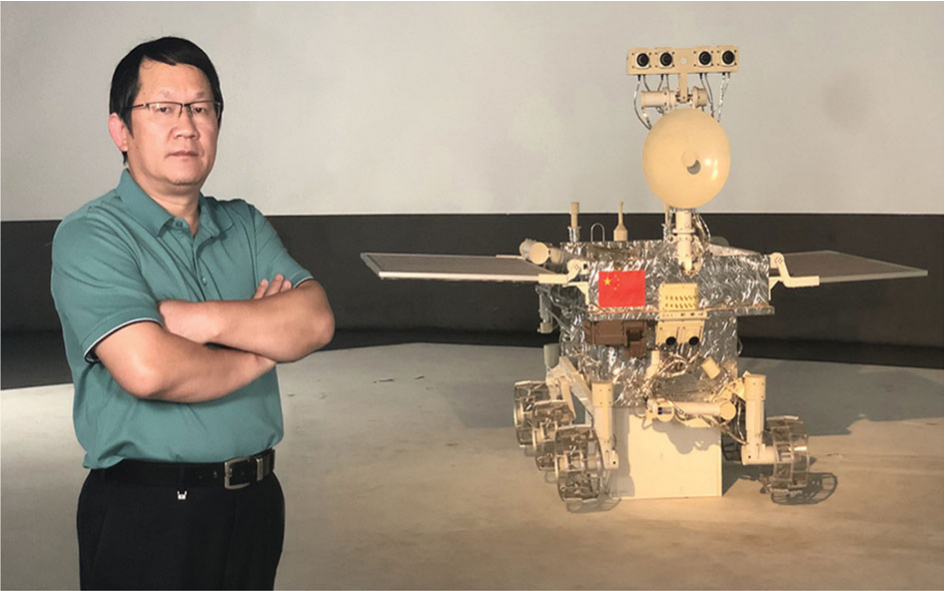
CLEP third phase Vice-Chief Engineer, CE-4 Ground Research and Application System Director Chunlai Li with a model of Yutu, the rover of CE-3 *(Courtesy of Chunlai Li)*.

**Wu:** Tens of thousands of people within more than 1000 units within China directly or indirectly contribute to CLEP. These include industrial units such as China Aerospace Science and Technology Corporation, China Aerospace Science and Industry Corporation and China Electronics Technology Group Corporation, research institutes of the Chinese Academy of Sciences, many universities, as well as other supporting units distributed around China. Coordination and cooperation among various units is crucial to the smooth implementation of CLEP.

**Ouyang:** From CE-1 to CE-4, the successes of these missions have proved that our organization system is highly effective and able to avoid big mistakes.

**Li:** The robotic part of CLEP consists of five subsystems: the satellite system, the rocket system, the launch site system, the tracking and control system, and the ground application system.

I am in charge of the ground application system, which can be considered as the scientific part of CLEP, so we also call this subsystem GRAS, the ground research and application system. Specifically, in the overall design stage, we set up scientific tasks and decide which instruments should be installed in which probes according to the characteristics and the space environments of these probes such as the orbiter, the lander, the rover and the returner. After the probes are launched, we give commands to the instruments to control their working states and to collect data. When the data are received, we perform basic data processing and analysis, and then release the data to the researchers. So, GRAS is responsible for the entire scientific chain of CLEP, from scientific task set-up to data analysis.

**NSR:** The CE-4 mission carried several international payloads. How will China promote international cooperation in its future space exploration projects?

**Li:** At present, cooperation between China and the US on space exploration is forbidden by the US government. But we can cooperate with other countries. Currently, China's CE-6 and the asteroid exploration project are publicly calling for payloads from the international academia. We have proposed a list of potential payloads. If any scientists are interested in the listed payloads and the related scientific projects, they can apply to provide certain payloads. If they have ideas not included in our list, we also encourage them to propose new payloads. We will actively promote such international cooperation in all our future projects.

**Figure 4:**
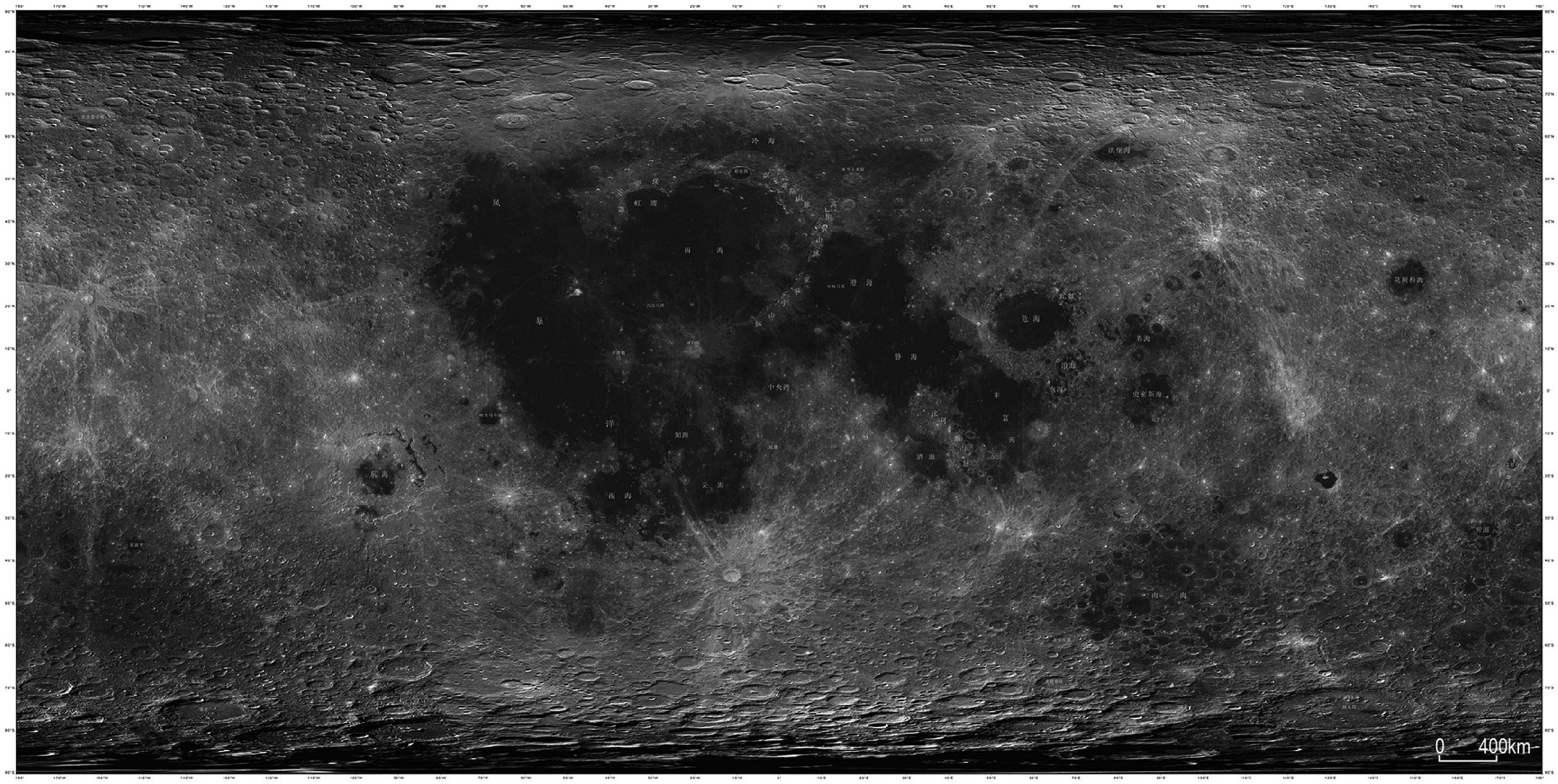
A lunar global topographic map produced using CE-2 image data. The spatial resolution is 7 m. The near-side of the Moon is presented in the middle and the far-side is presented on the right and left side of the map *(Courtesy of CLEP)*.

**NSR**: Private space companies such as SpaceX are developing fast. There are also many private space startups in China. How would these companies develop? Will they be a part of China's space exploration?

**Wu:** We welcome private companies to participate in China's lunar and deep space exploration and make necessary additions to the project.

Currently,there are a number of private rocket companies and even more satellite companies in China. Like SpaceX and other foreign private space companies, most founders and technicians of China's private space companies have worked previously in our national space system. The current situation is somewhat chaotic. I think after several years of adjustment and competition, some of the companies will stand out and have a bright future. China has begun to set rules for these companies in order to guide standardized operation and orderly competition in this field. I would be glad to see them develop well in the future.

GRAS is responsible for the entire scientific chain of CLEP, from scientific task set-up to data analysis.

—Chunlai Li

## CLEP'S PROMOTION OF CHINA'S INDUSTRY

**NSR**: Can CLEP benefit China's economy? For example, can we exploit lunar resources?

**Ouyang:** It is true that there are large amounts of resources on the Moon. For example, there is a large amount of titanium on the Moon. Also, the lunar soil is rich in ^3^He, which can be used as raw material for the fusion reaction, theoretically sufficient to meet the energy demand of all mankind for 10 000 years. But currently, we are not able to construct facilities on the Moon to extract ^3^He from lunar soil, and it is also impossible to take the soil back to the Earth for extraction. So the lunar resources are currently of no mining value and cannot bring economic benefits.

**Wu:** From another point of view, CLEP can promote the development of China's industry and thus bring economic benefits. For example, we developed the continuously variable thrust liquid engine for CLEP. This technology is currently only available in the US, Russia and China. We also developed cushioning pull rods, which were installed on the four legs of the CE-4 lander. They can change length automatically according to their stress conditions and ensure the smooth landing of CE-4. These rods can be used in many civil conditions. We also constructed a deep space tracking and control network with the largest antenna in China and thus laid the foundation for our further deep space explorations.

**NSR**: What is the localization level of CE-4 components? What are the gaps between China and the space powers such as the US and Russia?

**Wu:** About 96% of CE-4 materials and components are designed and produced in China, and the imported components are not vital parts. So the localization level of our space projects is rather high.

The space industries of the US and Russia have solid foundations, large scales and advanced technologies. We started late but developed fast. Now we have established a complete space industry and a complete product line. The world space industry started 100 years ago, and China started only 60 years ago. We have achieved so much within such a short time, so I believe that humans will definitely reach deeper space and do things we cannot imagine at this moment.

**Li:** For GRAS, the facilities such as antennas are all independently developed by ourselves. However, we do need a number of foreign instruments and equipment for the follow-up data and sample analysis.

## PERSONAL REFLECTIONS

**NSR**: Professor Ouyang and Professor Li, you both began your careers as geologists and later entered the field of space exploration. Is this a coincidence?

**Ouyang:** Planetary science can be considered as an extension of Earth science. It uses the concepts, theories and methods of Earth science to study other planets or natural satellites. There is a discipline called comparative planetary science, which uses the Earth as the standard and interpretsother planets or natural satellites by comparing them with the Earth. Many planetary scientists in China, Europe, Russia and the US majored in geology as graduate students.

**Li:** From this point of view, planetary science is somewhat different from astronomy. Currently, most astronomers study a celestial body as a point in space. They use spectra or other data to learn the overall properties of a star. But the planetary scientists want to know about the surface and the body of a celestial body in order to learn about its inner structure and evolutional history. Actually, astronomy is also beginning to pay more attention to the surfaces and bodies, which is the trend of future exploration.

**NSR**: How did you join CLEP? Have there been any memorable experiences during the project?

**Wu:** I have been engaged in the space industry since graduation from college and never changed my career direction. I love this job deeply. It is human nature to explore the unknown. Throughout these years, I have been deeply moved by the spirit of the Chinese space scientists and engineers. They work hard and cooperate well without considering personal gains and losses. It is this spirit that has made CLEP and other Chinese space projects possible.

**Ouyang:** The Soviet Union launched the first man-made satellite in 1957, opening the era of space exploration. In 1958, the United States and the Soviet Union started their lunar exploration projects. At that time, the People's Republic of China was newly established and was economically and technically ‘poor and blank’. I was then an associate doctoral candidate at the Institute of Geology of the Chinese Academy of Sciences and realized that we needed to prepare for the Chinese space exploration projects, which will surely be implemented in the future.

In 1958, I started to research on extraterrestrial materials such as meteorites and cosmic dust, as well as the effects of small celestial bodies impacting the Earth. These were the earliest studies of this kind in China. I led the research on the 1976 Jilin meteorite rain, which is the world's largest meteorite rain on record. In 1978, the then US President, Jimmy Carter, gifted an Apollo lunar sample to China, and I was lucky enough to take charge of the research work on this sample. During the same period, we made a comprehensive study of the space exploration plans of the United States and the Soviet Union, and preliminarily envisaged the future lunar exploration plans of China.

From 1993 to 2003, I actively promoted the systematic demonstration of CLEP. I led the study of ‘the Necessity and Feasibility of China's Lunar Exploration’, which was completed in 1995 and evaluated as ‘very necessary and fully feasible'. After that, we further designed the overall plan and phased tasks of CLEP. In 2003, the demonstration group with Professor Jiadong Sun (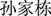) as the leader and myself as the vice-leader completed ‘The Comprehensive Project Report of China's First Lunar Exploration' with six appendices, which was reported to the State Council and approved for implementation. In 2004, I was appointed as the Chief Scientist of CLEP.

When the CLEP was announced in 2004, the public raised many questions such as ‘Why do we explore the Moon when there are so many things left to be explored on the Earth?' and ‘How much money will CLEP spend?’ I suddenly realized that I have the responsibility to clarify these questions. So I began to give lectures and speeches to the policy makers, the public servants, the students of primary schools, middle schools, high schools and the universities, as well as the public at large. During the 11 years from 2008 to 2018, I gave 617 public talks about

During the 11 years from 2008 to 2018, I gave 617 public talks about the Moon and the space exploration projects, covering more than 353 000 live audiences.

—Ziyuan Ouyang

the Moon and the space exploration projects, covering more than 353 000 live audiences. In the lectures broadcasted on the internet, the number of online audiences of each lecture reached 200 000 to 800 000. I also wrote more than 300 related education and public outreach articles and published 12 popular science books.

**Li:** After graduating from university, I spent three years involved in research on mineralogy. After that, I became a graduate student of Professor Ziyuan Ouyang, and started to conduct research on meteorites and celestial body impact events, including research about the Moon. Following Professor Ouyang, I participated in the determination of the scientific tasks of CLEP and took charge of the design and construction of GRAS. What impressed me most in CLEP is that space projects are systematic projects that need the strong collaboration of all the involved individuals and teams.

## Notes

Weijie Zhao is an NSR news editor based in Beijing; Chi Wang is the Director of the National Space Science Center, Chinese Academy of Sciences.

